# Neutrophil Extracellular Traps and Its Implications in Inflammation: An Overview

**DOI:** 10.3389/fimmu.2017.00081

**Published:** 2017-02-06

**Authors:** Vidal Delgado-Rizo, Marco A. Martínez-Guzmán, Liliana Iñiguez-Gutierrez, Alejandra García-Orozco, Anabell Alvarado-Navarro, Mary Fafutis-Morris

**Affiliations:** ^1^Physiology, Universidad de Guadalajara, Guadalajara, Mexico

**Keywords:** NETs, infectious diseases, autoinmmune diseases, autoinflammatory diseases, metabolic diseases

## Abstract

In addition to physical barriers, neutrophils are considered a part of the first line of immune defense. They can be found in the bloodstream, with a lifespan of 6–8 h, and in tissue, where they can last up to 7 days. The mechanisms that neutrophils utilize for host defense are phagocytosis, degranulation, cytokine production, and, the most recently described, neutrophil extracellular trap (NET) production. NETs are DNA structures released due to chromatin decondensation and spreading, and they thus occupy three to five times the volume of condensed chromatin. Several proteins adhere to NETs, including histones and over 30 components of primary and secondary granules, among them components with bactericidal activity such as elastase, myeloperoxidase, cathepsin G, lactoferrin, pentraxin 3, gelatinase, proteinase 3, LL37, peptidoglycan-binding proteins, and others with bactericidal activity able to destroy virulence factors. Three models for NETosis are known to date. (a) *Suicidal NETosis*, with a duration of 2–4 h, is the best described model. (b) In vital NETosis with nuclear DNA release, neutrophils release NETs without exhibiting loss of nuclear or plasma membrane within 5–60 min, and it is independent of reactive oxygen species (ROS) and the Raf/MERK/ERK pathway. (c) The final type is vital NETosis with release of mitochondrial DNA that is dependent on ROS and produced after stimuli with GM-CSF and lipopolysaccharide. Recent research has revealed neutrophils as more sophisticated immune cells that are able to precisely regulate their granular enzymes release by ion fluxes and can release immunomodulatory cytokines and chemokines that interact with various components of the immune system. Therefore, they can play a key role in autoimmunity and in autoinflammatory and metabolic diseases. In this review, we intend to show the two roles played by neutrophils: as a first line of defense against microorganisms and as a contributor to the pathogenesis of various illnesses, such as autoimmune, autoinflammatory, and metabolic diseases.

## Definition, Mechanisms, and Functions

In addition to physical barriers, neutrophils are considered part of the first line of immune defense. They can be found in the bloodstream, where they have a lifespan of 6–8 h, and in tissue, where they can last up to 7 days (1). They are the first cells of the immune system to migrate to a site of inflammation, where they play an important role in pathogen elimination and cytokine production (2).

The mechanisms that neutrophils undertake for host defense are phagocytosis, degranulation, cytokine production, and, the most recently described, neutrophil extracellular traps (NETs) production (3).

Neutrophil extracellular traps were discovered by Takei et al. (4) in 1996 as a pathway of cellular death different from apoptosis and necrosis. They were investigating the relationship of neutrophil activation and neutrophil death using phorbol-12-myristate-13-acetate (PMA); they observed morphological changes quite distinct to those that occur in apoptosis and necrosis, which led them to suggest that an alternative pathway of cell death could be taking place. First, they described the fusion of a multilobulated nucleus in neutrophils and the reduction of chromatin from its compact structure. The nuclear envelope then breaks down while cytoplasmic organelles remain intact. After 3 h, the extracellular membrane is disrupted in a mechanism dependent on reactive oxygen species (ROS) (4). Finally, in 2004, Brinkmann et al. (3) further detailed this process and named it *NETosis* (3).

Neutrophil extracellular traps are DNA structures released due to chromatin decondensation and spreading, and they thus occupy three to five times the volume of condensed chromatin. Several proteins adhere to NETs, including histones and over 30 components of primary and secondary granules (5), among which are components with bactericidal activity such as elastase, myeloperoxidase, cathepsin G (CG), lactoferrin, pentraxin 3, gelatinase, proteinase 3 (PR3), LL37, peptidoglycan-binding proteins, and others with bactericidal activity able to destroy virulence factors (6–9).

It is worth mentioning that chromatin and histones within the nucleus possess intrinsic antimicrobial activity. DNA acts as a chelating agent for cations due to its phosphodiester skeleton, rendering it capable of disrupting the external and internal membranes of *Pseudomonas aeruginosa* (10, 11). The antimicrobial effect of histones was observed in the 1950s by James Hisch, and H2A has been proposed as one of the most effective antimicrobial agents, particularly against *Escherichia coli, Shigella flexneri, Shigella sonnei, Salmonella enteritidis, Salmonella typhimurium, Klebsiella pneumoniae, P. aeruginosa, Staphylococcus albus*, and *Staphylococcus aureus*. In addition, recombinant H4 possesses antimicrobial activity against *S. aureus* and *Propionibacterium* (12). The antimicrobial effect of histones has been observed not only against bacteria but also parasites. Wang et al. observed that H2A and H2B reduced the replication of the *Leishmania* spp. promastigotes by up to 50% (13).

Three models for NETosis are known to date. *Suicidal NETosis*, with a duration of 2–4 h, is the best described model (14), even though its molecular processes are not fully understood (15). Stepwise, it starts with the activation of neutrophils through the recognition of stimuli, leading them to package and activate the NADPH oxidase (NOX) complex through protein kinase C (PKC)/Raf/MERK/ERK, as well as to increase cytosolic Ca^++^; these cations act as cofactors for peptidyl arginase deaminase 4 (PAD4), a nuclear enzyme that promotes the deamination of histones, thus modifying amino acids to allow the decondensation of chromatin by promoting the loss of the positive charges necessary for the interaction of histones with DNA (16, 17).

**Figure 1:**
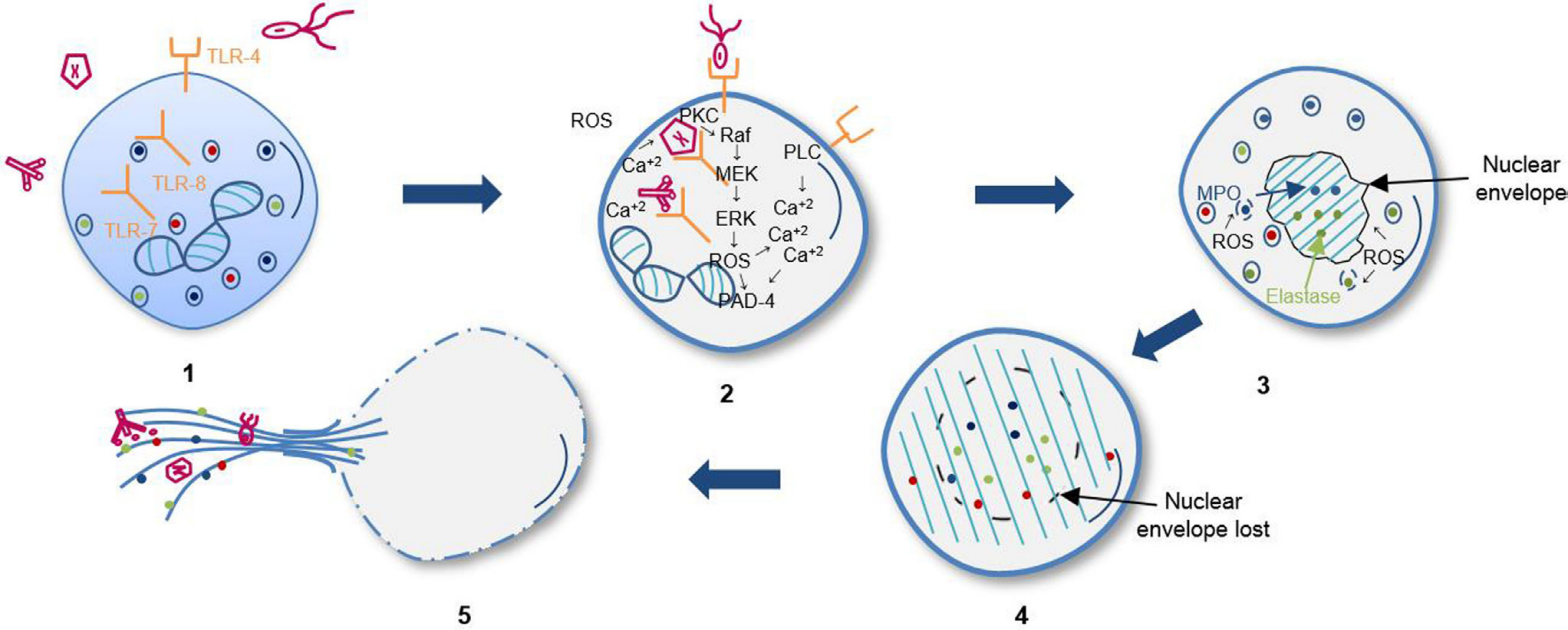
**Sequential steps of suicidal NETosis**. (1) Recognition of stimuli through receptors. (2) Activation of Raf/MEK/ERK kinases pathway and increase of cytosolic calcium that leads to gp91phox phosphorylation for activation of oxidase NADPH complex and subsequent reactive oxygen species (ROS) production. (3) Elastase and myeloperoxidase (MPO) translocation to nucleus from azurophil granules promoted by ROS and other yet unknown factors. Decondensation of chromatin and loss of the lobular shape of the nucleus. (4) Loss of nuclear and granular membrane, association of decondensed chromatin to cytoplasmic components. (5) Loss of plasmatic membrane and release of DNA as extracellular traps.

Reactive oxygen species behave as second messengers in suicidal NETosis by promoting the gradual separation and loss of the nuclear membrane, which disintegrates into small individual vesicles. Afterward, chromatin disperses throughout the cytoplasm, where it gets mixed with cytoplasmic proteins and granule toxins. NET formation is dependent on elastase and myeloperoxidase transport all the way from granules to the nucleus (18). Finally, chromatin is released outside the cell through membrane pores and cellular lysis. Suicidal NETosis is dependent on ROS for histone citrullination by PAD4, which allows chromatin decondensation (16, 17, 19), finally releasing the DNA as extracellular traps (ETs) (Figure 1) (20, 21).

**Figure 2:**
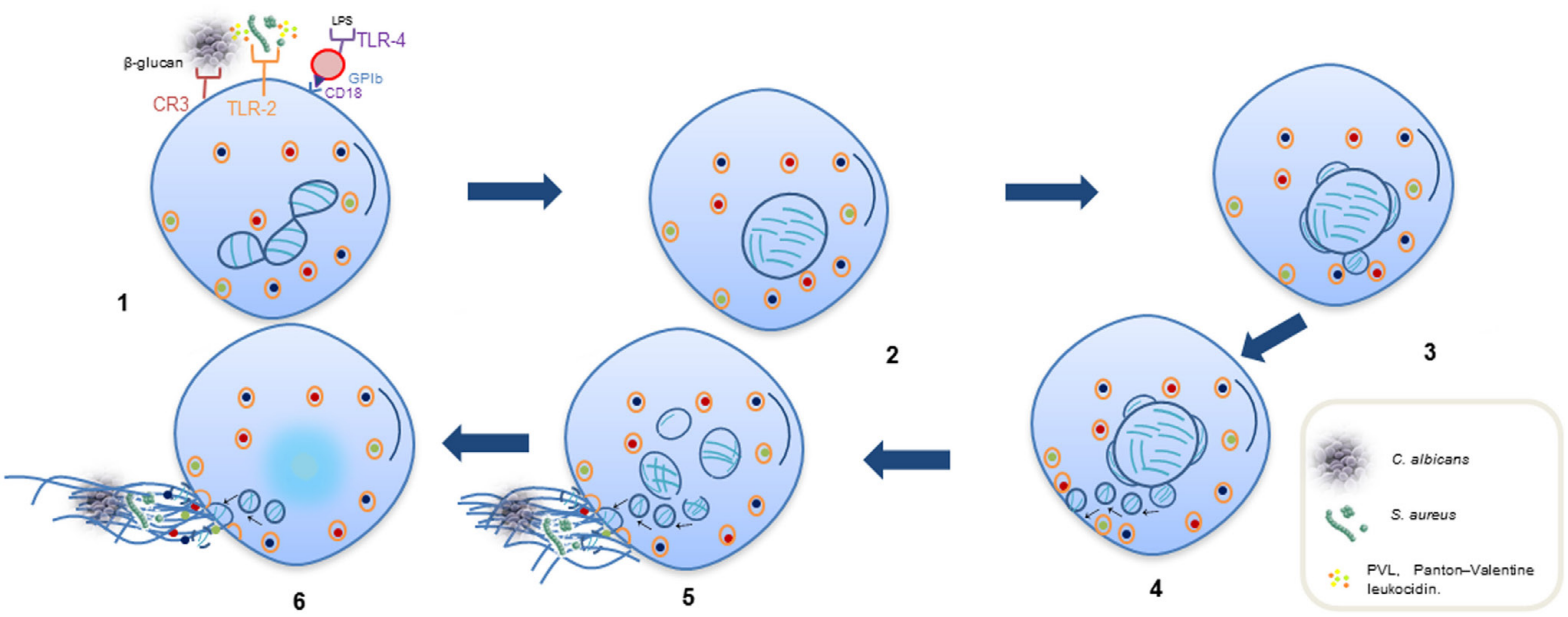
**Sequential steps of vital NETosis**. (1) Recognition of stimuli through receptors. (2) Loss of the lobular and multinucleated shape of the nucleus. (3, 4) Separation of external and internal nuclear membranes and budding of vesicles. (5) Vesicles in cytoplasm containing DNA filaments in form of pearl strings, approaching of dense cytoplasmic granules toward intact plasmatic membrane. (6) Release of DNA as extracellular traps released through a small area in cell surface; some cytoplasmic granules also fuse to plasmatic membrane and are released into extracellular space to associate with DNA.

In *vital NETosis*, neutrophils release NETs without exhibiting a loss of nuclear or plasma membrane within 5–60 min, and it occurs independently of ROS and the Raf/MERK/ERK pathway. This process consists of the release of nuclear DNA through three morphological changes: (a) nuclear envelope growth and vesicle release, (b) nuclear decondensation, and (c) nuclear envelope disruption (14, 22–24). This type of NETosis is triggered by the recognition of stimuli through toll-like receptors (TLRs) and the complement receptor for C3 protein (25–27). Furthermore, interaction between glycoprotein Ib in platelets with β2 integrin (CD18) in neutrophils may induce NET formation by the activation of ERK, PI3K, and src kinases (Figure 2) (28). After release of the nucleus, these neutrophils are still able to phagocytose pathogens, and their lifespan is not affected by DNA loss (24).

Finally, Yousefi has described another type of vital NETosis dependent on ROS, in which mitochondrial DNA is released instead of nuclear DNA; this process results in NET formation from 80% of neutrophils within 15 min through recognition of C5a or lipopolysaccharide (LPS) (29).

This review will focus on NET participation in microbial infections, autoimmunity, and metabolic disorders. A detailed description of the mechanisms involved in NET formation is beyond the scope of this work, but additional reviews can be found elsewhere (30). However, it is important to highlight high-mobility group box 1 (HMGB1) protein-expressing platelets as major endogenous inducers of NET formation, not only during infectious processes but also in sterile inflammation (31, 32).

## NETs and Pathogens

There are several inflammatory processes triggered by the presence of bacteria, viruses, parasites, and fungi. Since bacteria and their metabolic products were first described to stimulate NET formation, the mechanisms for both the initiation and evasion of NETs by pathogens have been intensively studied (Table 1).

**Table 1:** **Microbe inducing NETosis**.

Microbes	Microbe peptides modulating neutrophil extracellular traps (NETs)	Pathologies	Effects in NETosis	Types of NETosis	References
**Bacteria**					

*Staphylococcos aureus*	Leukotoxin GH and Panton–Valentine leucocidin	Osteomyelitis, endocarditis, bacteremia, and gastroenteritis	Reactive oxygen species (ROS)-independent induction with nuclear DNA liberation, toll-like receptor (TLR)2, and C3 dependent	Vital	(14, 27)

*Streptococcus pneumoniae*	EndA and α-enolase	Pulmonar influenza, chronic obstructive pulmonary disease, pneumococcal pneumonia, and emphysema	ROS-independent induction	Suicidal	(33)

*Escherichia coli*	ND	Enteritis, urinary infeccions, meningitis, and sepsis	Platelet free induction, TLR4 independent, and ROS dependent	Absence of platelets: suicidal	(34, 35)

*E. coli*			Induction with platelet presence, TLR4 dependent, and ROS independent	Presence of platelets: vital	(26, 28, 36)

*Clostridium difficile*	ND	Diarrhea and pseudomembranous colitis	Induction	ND	(37)

*Shigella flexneri*	IcsA and IpaB	Dysentery	Induction	Suicidal	(3)

*Salmonella typhimurium*	ND	Infectious gastrienteritis	Induction	Suicidial	(3)

*Yersinia*	Yops proteins and invasin protein	Yersiniosis, acute enteritis, and enterocolitis	ROS-dependent induction, PI3K signaling, and β-integrin pathway	Suicidal	(38, 39)

*Mycobacterium tuberculosis*	Adhesins, heat shock protein 72, and ESAT/6	Tuberculosis	Phagocytosis-dependent induction, ROS, and elastase	Suicidal	(40, 41)

*Vibrio cholerae*	Dns and Xds	Cholera	ROS-dependent induction	Suicidal	(42)

*Lactobacillus rhamnosus* GG	P40 and P75	–	Inhibition	–	(43)

**Virus**					

Influenza virus	ND	Influenza A H1N1	ROS- and peptidyl arginase deaminase 4-dependent induction	Suicidal	(44)

Dengue virus serotype 3	ND	Dengue	NETs inhibition and Glut-1 decreasing glucose captation	–	(45)

HIV	ND	Acquired immune deficiency	ROS-dependent induction	Suicidal	(46)

Respiratory syncytial virus	Fusion protein	Acute bronchitis	F protein induction across TLR4, ROS, ERK, and MAPK p38	Suicidal	(47)

**Yeast**					

*Candida albicans*	–		Complement receptor 3- and fibronectin-dependent induction, and ROS independent	Vital	(25)

*Asperguillus fumigatus*	RodA	Invasive aspergillosis	ROS-dependent induction. *A. fumigatus* spores containing RodA do not induce NETs	Suicidal	(48, 49)

*Cryptococcus neoformans*	–	Cryptococcal meningitis	ROS and NETs inhibition	–	(50)

**Parasites**					

*Plasmodium falciparum*	Antihemostatic agaphelin	Malaria	Induction through *P. falciparum* and inhibition through agaphelin	Suicidal	(51, 52)

*Toxoplasma gondii*	–	Toxoplasmosis	MEK–ERK-dependent induction	Suicidal	(53)

### Bacteria

#### *Staphylococcus* *aureus*

*Staphylococcus aureus* is a Gram-positive bacterium that mainly resides in the wet squamous epithelium of the anterior nasal cavity (54) and is known as a “super bacterium” due to its capacity to evade the immune system and resist antibiotic treatment (55). It causes pathologies such as osteomyelitis, endocarditis, bacteremia, and gastroenteritis, which are related to a severe inflammatory response (56).

In classic assays performed by Brinkmann in 2004, *S. aureus* was first used as a stimulus for unleashing NETosis (3). In 2010, when inducing NET formation and studying its molecular mechanisms, Pilscek et al. discovered that *S. aureus* led to a notably faster NETosis that was independent of ROS, which they named “vital NETosis” (14).

*Staphylococcus aureus* secretes several virulence factors that allow it to evade the host immune system, among which are Panton–Valentine leucocidin (PVL), leukotoxin GH (LukGH), leukotoxin DE, gamma-hemolysin, and N-terminal ArgD peptides, of which LukGH and PVL promote NETs through an oxidative pathway-independent mechanism (14, 57, 58).

Bacterial invasion promotes NET formation to immobilize pathogens and hinder their spread. This innate immune response is supported by macrophages through phagocytosis and cytokine production. Thammavongsa et al. have reported that *S. aureus* may have a cytotoxic effect on macrophages through these NETs, since NET incubation with nucleases and adenosine synthases derived from this bacterium promotes the formation of deoxyadenosine, which is capable of inducing cell death (59).

#### *Streptococcus* *pneumoniae*

*Streptococcus pneumoniae* is a Gram-positive bacterium that can be found normally in the human respiratory tract (60), and its role in NET induction has been established (3).

It has been found that during pulmonary infection, similar to *S. aureus, S. pneumoniae* is able to produce virulence factors (endA) that degrade DNA and allow it to escape from NETs even after bacteria have been caught within, promoting bacterial dissemination from the upper respiratory tract to the lungs and then to the bloodstream (61–63).

Neutrophil extracellular trap release has also been implicated in the development and complications of respiratory diseases due to secondary infections such as chronic obstructive pulmonary disease, pneumonia, and emphysema (64–66). Neutrophils, when excessively recruited to lung tissue in response to infection, disrupt microcirculation and induce more NETosis in pulmonary alveoli. Furthermore, patients with pulmonary dysfunction show higher levels of extracellular DNA than patients with mild lung disease, showing that NETs participate in airflow obstruction and perpetuate chronic inflammatory responses (67, 68).

#### *Escherichia* *coli*

*Escherichia coli* is a Gram-negative bacterium that colonizes the human gastrointestinal tract at birth (69), and it is the most abundant facultative anaerobe among the host microbiota. It has been implicated in pathologies such as enteritis, urinary infections, meningitis, and sepsis (70).

Finally, Kambas et al. found that NETs are significantly induced when neutrophils are stimulated with the serum of patients with septic shock triggered by *E. coli*, probably through activation of TLRs or complement receptors for C3 or C5a (34).

It has been reported in several studies that septicemia is aggravated by NETs and their components (71–73), since their degradation with DNases along with antibiotic treatment attenuates tissue damage (74).

Neutrophils are capable of discriminating between LPS from different pathogens and strains in order to induce NET formation and release tissue factor (TF), a thrombogen that has been implicated in systemic inflammatory responses mediated by activation of the coagulation system that characterizes septic processes (36, 75).

*Escherichia coli* strain Afa/Dr has been studied in infantile diarrhea, where it has been shown to promote the activation of several signaling pathways of epithelial cells, especially those involved in functional and structural injury to the intestinal barrier (76). Regarding the relationship of this process and NETs, Marin-Esteban et al. showed that these structures are able to capture, immobilize, and eliminate bacteria. However, when neutrophils are cocultured with the epithelial cell line CaCo-2/TC7 and bacteria, the former produce NETs and harm the epithelial cells. For this reason, they suggest that NETs may be involved in injury to the intestinal epithelium as well as other intestinal inflammatory diseases (35).

Concentrations of the antimicrobial peptide (AMP) LL37 by cells of the urinary tract are not able to eliminate infections caused by *E. coli*. Nevertheless, LL37 derived from recruited neutrophils significantly decreases bacterial colonization (77). Since LL37 is associated with NETs (3) and promotes their formation and stability (78), it seems likely that it plays a relevant role in pathogen elimination by cooperating with NETs, although this has not yet been proven.

#### *Clostridium* *difficile*

*Clostridium difficile* causes diarrhea and pseudomembranous colitis in humans, generally due to the abuse of antibiotics (79) that severely harm the host resident microbiota, leading to dysbiosis (80).

*Clostridium difficile* is a normal component of the human microbiota, and its competition for nutrients with other resident species normally prevents its excessive reproduction (81). However, when the microbiota becomes altered, nutrient availability is increased along with the diminished production of secondary biliary acids, which allows for *C. difficile* colonization of the gut (82, 83).

*Clostridium difficile* is able to translocate through the ability of its enterotoxins to cause the loss of tight junctions. When *C. difficile* comes into contact with cells of the gut-associated lymphoid tissue, it promotes the production of proinflammatory cytokines and chemokines such as interleukin 1 beta (IL-1β), IL-8, and CXCL5 to promote the recruitment of neutrophils (84, 85).

Neutrophils not only reduce the function of microbial toxins by secreting AMPs and elastase but also produce NETs, which may act to cover the injured areas of the intestinal epithelium to effectively hinder *C. difficile* dissemination (37).

#### *Shigella* *flexneri*

*Shigella flexneri* is a Gram-negative enteropathogenic bacterium usually acquired by the ingestion of contaminated food and beverages and whose infection may cause dysentery in the host. *Shigella* can traverse the intestinal lumen through M cells; once there, it infects epithelial cells and may propagate horizontally. As a response, nuclear factor kappa B (NF-κB) is activated in infected cells, which produce IL-8 to attract the migration of neutrophils to infected tissues, where neutrophil-derived elastase degrades microbial virulence factors (86, 87).

Brinkmann et al. showed that *S. flexneri* is trapped within NETs *in vitro* and tested the ability of DNA-associated elastase to degrade the virulence factors IcsA and IpaB (3).

Perdomo et al. showed that, for *Shigella* pathogenesis, *in vitro* neutrophil transmigration is required for *Shigella* invasion in zones with intense neutrophil infiltration (88).

#### *Salmonella* *typhimurium*

*Salmonella* is a genus of facultative anaerobic intracellular bacteria. Even though many species of this genus can be found in the gut microbiota, *S. typhimurium* is a leading cause of infectious gastroenteritis. After colonizing the gut, it can enter into enterocytes, M cells, dendritic cells (DCs), and, finally, into macrophages upon reaching the submucosa. *S. typhimurium* replicates inside phagosomes where it may express several virulence factors: adhesins, flagella, fimbriae, and T3SS (89). It is also capable of using a superoxide dismutase known as SodCl to counteract the activity of ROS in the phagosome of leukocytes (90, 91).

*Salmonella typhimurium* has been shown to stimulate NETs by Brinkmann et al. They have also shown that it is effectively trapped and eliminated by components of NETs, including granular proteins and H2A histone (3).

#### *Yersinia* 

*Yersinia enterocolitica* is the causal agent of yersiniosis, acute enteritis, and enterocolitis. It invades the epithelium and translocates to Peyer’s patches and affects tight junctions by decreasing occludin and claudins 5 and 8 (92). All three pathogenic species of *Yersinia*, namely, *Y. pseudotuberculosis, Y. enterocolitica*, and *Y. pestis*, mainly aim to translocate their effector proteins (known as Yops) into neutrophils, macrophages, and DCs. Additionally, *Y. pestis* inhibits the oxidative burst of neutrophils in order to promote its own intracellular survival using Yops proteins (93).

In 2015, Möllerherm et al. proved that serotypes 0:3, 0:8, and 0:8 of *Y. enterocolitica* induce NETs *in vitro* within 1 h of incubation. However, NET induction was diminished as the incubation time increased, suggesting that NETs may be degraded by the effects of Ca^++^- and Mg^++^-dependent nucleases (38).

*Yersinia pseudotuberculosis* employs a specialized protein called invasin to breach the intestinal epithelium. Invasin is a highly adhesive protein that mediates *Y. pseudotuberculosis* binding to the β1 integrins of M cells; however, this binding induces ROS production and NET formation (39).

#### *Mycobacterium* *tuberculosis*

*Mycobacterium tuberculosis* is an obligate aerobic bacillus that causes tuberculosis. It is one of the most successful intracellular pathogens regarding its strategies for evasion of immune system. It primarily infects the respiratory system, but it may also affect other organs. Its cell envelope contains adhesins and, in contrast to other pathogenic bacteria, it does not produce toxins. It uses phagocytes for replication as well as a way to disseminate throughout the host organism.

It has been shown that different genotypes induce NETs when they are cocultured with neutrophils. Even though NETs effectively trap and hinder the dissemination of mycobacteria, NET-derived components are unable to kill them (40).

A mechanism of immune evasion that *M. tuberculosis* utilizes is to augment the apoptosis of neutrophils to stop them from creating granulomas, which are structures composed of immune cells in response to primary infection, to contain this bacillus (94). Macrophage efferocytosis of apoptotic neutrophils leads the immune response toward a proinflammatory pole. Heat shock protein 72 has been found in apoptotic and necrotic cells (95) and in DNA within NETs, where it is also necessary for the elimination of *M. tuberculosis* (41).

#### *Vibrio* *cholera*

*Vibrio cholerae* is a Gram-negative bacterium widely recognized because of cholera pandemics provoked by the O1 and O139 serogroups. This bacterium can be found in the human gastrointestinal tract and in aquatic environments; concordantly, infections usually follow the ingestion of contaminated seafood and water. When *V. cholerae* reaches the gut, it secretes cholera toxin as well as adhesins, hemagglutinin, proteases, and hemolysins. Finally, *V. cholerae* induces the production of cytokines as well as neutrophil recruitment to the gut (96).

It has been reported that *V. cholerae* induces NETs upon *in vitro* contact with neutrophils. However, *V. cholerae* secretes the nucleases Dns and Xds as an evasion mechanism that allows it to escape from NETs, thus allowing it to continue the infectious process. Therefore, NETs have not been shown to be a protective mechanism for containing infection by *V. cholerae* (42).

#### *Lactobacillus* *rhamnosus*

*Lactobacillus rhamnosus* is considered an important probiotic for the microbiota. It is able to adhere to the intestinal epithelium and to resist gastric acid and bile (97).

It is a Gram-positive bacterium that has been primarily studied for its capacities to restore the intestinal barrier, as it reduces the epithelial injury caused by ulcerative colitis (UC) and Crohn’s disease (CD) (98).

*Lactobacillus rhamnosus* GG modulates the immune response and intestinal microbiota by stimulating TLRs on immune cells (99).

It has been shown that *L. rhamnosus* GG inhibits NET formation induced by either microbes (*S. aureus* and *E. coli*) or chemicals (phorbol 12-myristate 13-acetate, better known as PMA), probably due to its antioxidant activity and yet unknown secreted proteins (43).

### Viruses

#### Influenza

Influenza A virus is known for killing over 50 million people in 1918 and, recently, for the 2009 pandemics responsible for 18,000 deaths around the world. Influenza pathology is characterized by excessive neutrophil recruitment to the lungs, which is facilitated by CXCR2. Influenza A-stimulated NETs are dependent on PAD4 (100). α-Defensin-1 associated with NETs inhibits virus replication through the blockade of the PKC pathway.

Another component of the bactericidal nets stimulated by influenza A virus is LL37, which has been shown to increase NET production in response to this pathogen *in vitro* (44). Additionally, arginine-rich H3 and H4 histones are important for viral aggregation and neutralization. Incubation of influenza A virus with H4 was shown to lead to a significant decrease in viral replication in epithelial cells; by contrast, H4 was inactivated when incubated with the pandemic strain H1N1, which may highlight its importance in response to this pathogen (101).

The downsides of excessive neutrophil infiltration include injury to tissues mediated by AMPs and extensive NETs in the alveolar capillaries (67, 68).

#### Dengue

Dengue virus (DENV) is a single-stranded RNA virus that belongs to the Flaviviridae family. Infection with any of the dengue serotypes (1–4) has a range of effects, from mild fever to severe dengue, formerly known as dengue hemorrhagic fever (45). The incidence of dengue infections has increased in recent years (102); therefore, it is necessary to understand the mechanisms of host defense used to fight this pathogen.

Neutrophil extracellular traps are able to restrain infections by trapping viruses within their structures. However, it has been demonstrated that rather than stimulating NETs, DENV-2 inhibits them *in vitro*. Moreno-Altamirano et al. have observed an 80% reduction in PMA-stimulated-NET formation by neutrophils following previous incubation with DENV-1. This inhibition was caused by the disruption of Glut-1-mediated glucose uptake (45), a metabolic requirement for NET release (103).

#### Human Immunodeficiency Virus 1

Human Immunodeficiency Virus 1 (HIV-1) is a virus with tropism for the immune system. There are over 35 million people currently infected, with approximately 2 million acquiring the infection every year (104). It has been shown that CD4, along with CCR5 and CXCR4, acts as the receptor for virus entry, which allows not only for the infection of CD4 T cells but also antigen-presenting cells such as macrophages and DCs. However, most serum plasma is derived from activated T cells, where viral replication is quick and efficient (105).

Neutrophils recognize HIV-1-derived nucleic acids through TLR7 and TLR8. Afterward, they release ROS in order to induce NET formation. These structures may trap, contain, and eliminate HIV through the action of myeloperoxidase and α-defensins, to both of which antiviral activity has been attributed. HIV, as a mechanism of evasion, promotes IL-10 production by DCs, thus inhibiting ROS and NET release (46).

#### Respiratory Syncytial Virus

Respiratory syncytial virus (RSV) is the leading cause of hospitalization in 1-year-old infants (106); thus, it represents one of the most important pediatric infections. RSV causes acute bronchitis, mucosal and submucosal edema, and luminal occlusion by cellular debris formed from epithelial cells, macrophages, fibrin strands, and mucin. RSV also infects DCs and reduces their antigen-presenting capacity to activate T cells (107).

To generate NETs *in vitro*, RSV may stimulate neutrophils, and in turn, these have been shown in samples of bronchoalveolar lavage fluid from patients with severe disease of the lower respiratory tract caused by RSV. NET formation prevents RSV dissemination but seems unable to kill the virus (108). Additionally, RSV F protein is also able to induce NETs *via* TLR4. Despite these NETs acting as viral reservoirs, their presence may aggravate inflammatory symptoms and promote luminal occlusion with structures composed of mucus and DNA (47).

### Fungi

#### *Candida* *albicans*

*Candida albicans* is usually found colonizing the mucosa, skin, and oral cavity in healthy individuals, causing disease only in immunocompromised subjects, such as patients with pancreatitis or renal insufficiency, patients on antibiotic treatment or with a central venous catheter, and in patients following gastrointestinal surgery. *C. albicans* morphologically changes from yeasts to hyphae producing several virulence factors such as Als3 and Ssa11 invasins, turning itself into an invasive pathogen. Epithelial cells and macrophages that recognize *C. albicans* respond by releasing chemokines that attract neutrophils (109, 110). Neutrophils are able to trap and eliminate *C. albicans* in either its yeast or hyphal form by releasing NETs (111). To achieve a fast NETosis response, the β-glucan on hyphae must be recognized by complement receptor 3, and fibronectin, a component of the extracellular matrix, must be present. These elements are required for homotypic cellular aggregation supported by NETs but are independent of ROS production (25).

#### *Aspergillus* *fumigatus*

*Aspergillus fumigatus* is part of the human microbiota in healthy subjects. In immunosuppressed individuals, it is responsible for invasive aspergillosis, a leading mycotic infection by both prevalence and mortality rate in patients with chronic granulomatous disease (112). Infection occurs through the inhalation of spores, which instead of being eliminated by immune system cells, take up residence in the respiratory tract and alters their morphology from yeast to hyphae, infecting the lungs and leading to pneumonia and infection of other organs. Similar to *C. albicans*, it produces invasins that allow it to adhere to host cells (113, 114).

Release of NETs induced by *A. fumigatus in vitro* requires activation of NOX (48). Furthermore, p46^−/−^ mice cannot form NETs (115). Even though NETs are necessary for the capture and elimination of *A. fumigatus* hyphae, these are not induced by spores due to the presence of RodA in the spore cell wall (49).

#### *Cryptococcus* spp.

*Cryptococcus neoformans* is an opportunistic pathogenic yeast. Infection develops after spores are inhaled and enter into the alveolar space, where they remain latent until immunological disequilibrium occurs and leads to cryptococcosis and meningoencephalitis (116).

*Cryptococcus neoformans* possesses a capsular polysaccharide that confers it with the ability to regulate the host immune system. In particular, it is able to modulate NET production. Neutrophils incubated with strains whose capsules contained glucuronoxylomannan (GXM) and galactoxylomannan were not efficient producers of either ROS or NETs, even after PMA stimulus. When neutrophils were incubated with strains without capsular GXM, NETs were effectively produced; however, ROS were not observed. Thus, capsular GXM improves virulence by mediating resistance to NETs. Finally, the microbial activity of NET-associated AMPs, such as elastase, myeloperoxidase, collagenase, and histones, is required to kill this pathogen (50).

### Parasites

#### *Plasmodium* *falciparum*

*Plasmodium falciparum* is the causal agent of malaria, also known as paludism. It severely affects children under 5 years old, who represent 90% of deaths related to this disease (117). Malaria is a hematological disease transmitted by mosquitoes. *Plasmodium* spp. infect erythrocytes, which induces the production of inflammatory cytokines; these suppress erythropoiesis and lead to anemia. Invasion is mediated by proteins on infected erythrocytes that promote their adhesion to the vascular endothelium present in tissue and organs and induce an inflammatory response and coagulation (118, 119).

This infection process causes vascular damage, lesions on endothelial cells, and activation of platelets, monocytes, and neutrophils. Neutrophils release NETs, and these structures can be found in the circulation of children infected with *P. falciparum* adhering to erythrocytes and parasites. Additionally, α-dsDNA antibodies are found in these patients and may participate in the development of this pathology, aggravating the immune response and autoimmune processes (51). On the other hand, the glands of infected mosquitoes produce the antihemostatic agaphelin, which is able to inhibit neutrophil chemotaxis, blockade platelet aggregation mediated by cathepsin/elastase, and attenuate neutrophil-induced coagulation (52).

#### *Toxoplasma* *gondii*

*Toxoplasma gondii* is the causal agent of toxoplasmosis, which infects over a third of the population worldwide as a result of the ingestion of contaminated food.

Infection by *T. gondii* induces neutrophil recruitment to the infected site (120). Accordingly, in a murine model of intranasal infection, neutrophils limit the dissemination of this pathogen by trapping it and killing it in NETs, thus demonstrating that active invasion is not necessary for NET formation. This observation was subsequently shown in humans, further showing that NET formation is MEK–ERK dependent (53).

## NETs and Autoimmunity

Neutrophils and NETs play a dual role in host homeostasis. They both protect hosts from infectious diseases; however, they also cause pathologic alterations, as is the case in autoimmune and autoinflammatory diseases.

### Psoriasis

Psoriasis is a chronic inflammatory disease that affects the skin and is characterized by a complex immune response, since cellular, molecular, and vascular components participate in the perpetuation of the inflammatory process. To date, T helper 1 (Th1) and Th17 lymphocytes are considered as the sole regulatory cells of the immune response in psoriatic lesions (121, 122). However, Lin et al. have shown that IL-17 is abundantly produced by neutrophils and mast cells, both of which are found in the cellular infiltrates of psoriatic plaques (123). Initially, interferon alpha (IFN-α)- and tumor necrosis factor alpha (TNF-α)-secreting plasmacytoid dendritic cells (pDCs) are activated through TLRs upon the recognition of LL37–nucleic acid complexes released by damaged keratinocytes to secrete their cytokines. Initially, pDCs are activated through TLRs upon the recognition of LL37–nucleic acid complexes released by damaged keratinocytes, and consequently, the pDCs release IFN-α and TNF-α (124). These cytokines might favor the activation of DCs and macrophages and their production of IL-23 and IL-1β, which in turn induce the activation and production of IL-17, TNF-α, CXCL2, chymase, and tryptase by mast cells through mast cell extracellular trap (MCET) formation (125). Additionally, these mediators promote neutrophil migration toward the epidermis, where they may become activated by IL-23 and IL-1β and produce NETs. Together, both neutrophils and mast cells secrete IL-17 and other proinflammatory mediators to amplify neutrophil migration, which will then contribute to the formation of Munro’s abscesses (126).

**Figure 3:**
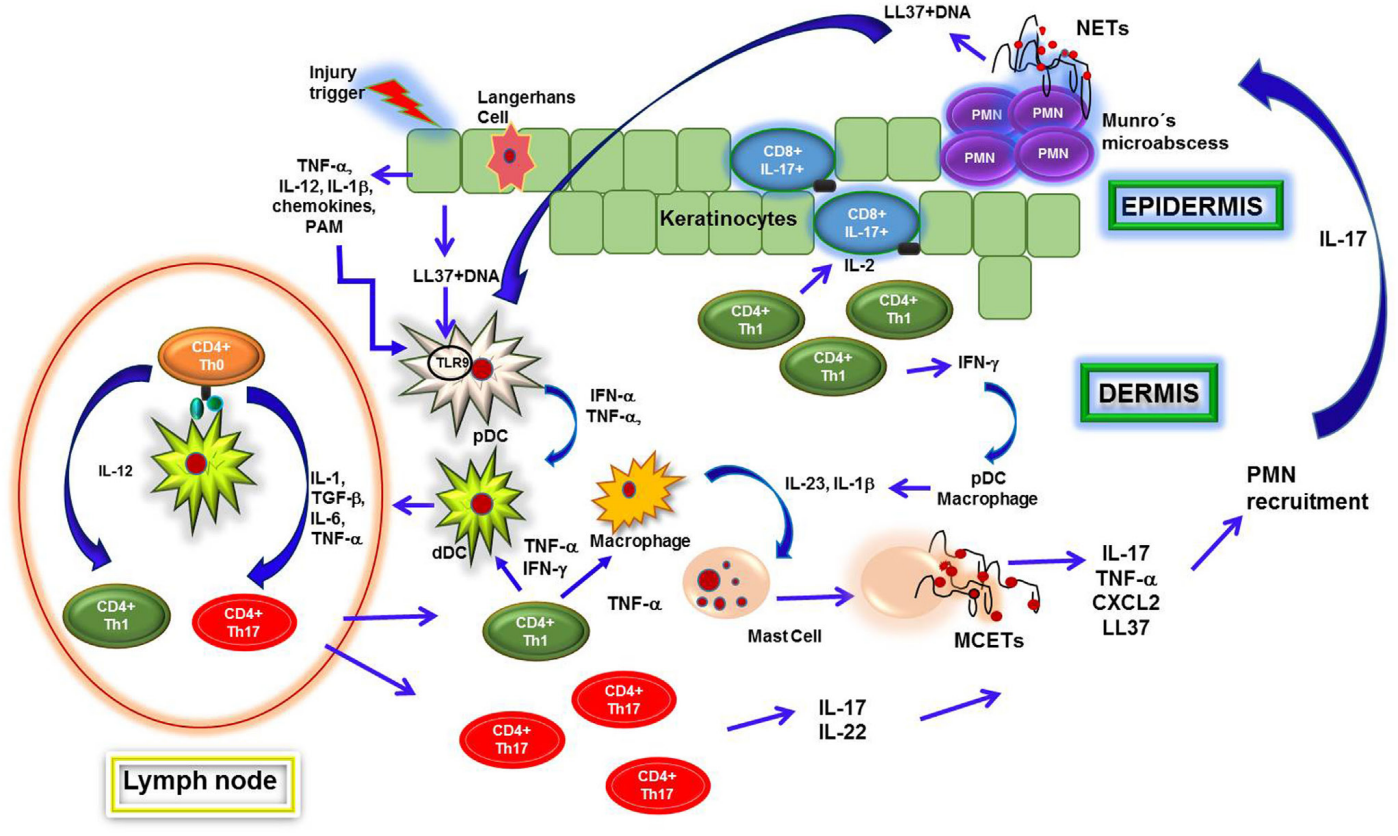
**Neutrophil extracellular traps (NETs) formation in psoriatic plaques**. Psoriatic lesions in patients are originated after injuries in skin, which induce keratinocytes to release proinflammatory cytokines and antimicrobial peptides. Such molecules, like cathelicidin LL37, form complexes upon binding to DNA that activate plasmacytoid dendritic cells (pDCs) through interaction with toll-like receptor 9 (TLR9), leading them to secrete interferon alpha (IFN-α) and tumor necrosis factor alpha (TNF-α). These cytokines regulate dermal dendritic cells (dDCs) activation, which migrate to regional lymphoid nodes to present autoantigens to naïve CD4 T (Th0) cells. Subsequently, T cells differentiate into T helper 1 (Th1) or Th17 and migrate toward dermis, where they secrete IL-2, IFN-γ, TNF-α, IL-22, and IL-17 that contribute to recruitment and activation of macrophages, dDCs, and mast cells. These cells synthesize IL-23 and interleukin 1 beta (IL-1β) and thus induce release of mast cell extracellular traps (MCETs). Consequently, mast cells intracellular content is released along with IL-17 and other cytokines, which induce neutrophil infiltration into epidermis and formation of Munro’s microabscesses. Neutrophils encounter high concentrations of IL-23 and IL-1β in this microenvironment, which turn them susceptible to release NETs. Through NETs formation, they too secrete cellular contents including IL-17, thus amplifying the inflammatory process and increasing cells recruitment and keratinocytes activation. Finally, NETs are important sources of LL37–DNA complexes and IL-17, both of which activate pDCs and keratinocytes that, in turn, produce IFN-α and LL37, respectively. Th1 cells induce activation of T CD8 IL-17-producing cells in epidermis and pDCs within dermis, perpetuating the inflammatory environment in psoriatic lesions.

On the other hand, IL-17 in keratinocytes increases the expression of LL37 and defensins such as beta-defensin 2 (HBD-2), S100A7, S100A8, and S100A9 (127, 128), which mediate cellular infiltration and both MCET and NET formation. In this context, ET-derived DNA–LL37 complexes are usually generated. These complexes may lead to pDC activation and the consequent production of IFN-α, further promoting NETosis and inflammation in psoriatic lesions even in the absence of infection (124) (Figure 3).

### Systemic Lupus Erythematosus

Systemic lupus erythematosus (SLE) is an autoimmune disease characterized by immune complexes and high levels of IFN-α induced by the presence of autoantigens due to a failure to eliminate products derived from apoptotic or necrotic cells (129, 130). Loss of tolerance toward self-antigens leads to the activation of autoreactive B cells and the production of autoantibodies against nucleic acids and AMPs, which are released by infiltrating neutrophils that undergo NETosis in the skin and kidneys of patients with SLE. The generated immune complexes may deposit in different tissues, resulting in injury and inflammation, provoking mainly cutaneous lesions, nephritis, and cardiovascular disease (131, 132). Furthermore, opsonized autoantigens induce pDCs to secrete IFN-α, also known as an “IFN-α signature,” and induce neutrophils to form NETs (133, 134).

In patients with SLE, nucleic material derived from dead cells accumulates due to the failure of its elimination. These self-antigens are presented to autoreactive B cells in germinal centers in secondary lymphoid organs by follicular dendritic cells, thus generating autoantibodies against cellular components derived from NETosis and apoptosis (134, 135). The production of immune complexes activates the complement system and induces inflammation, vascular injury, thrombosis, and brain damage. Immune complexes are internalized by pDCs through type II Fcγ receptor-mediated endocytosis; afterward, they associate with TLR7 and TLR9 on endosomes, which leads to the activation of IFN-α-secreting pDCs and additional formation of NETs (136–138).

**Figure 4:**
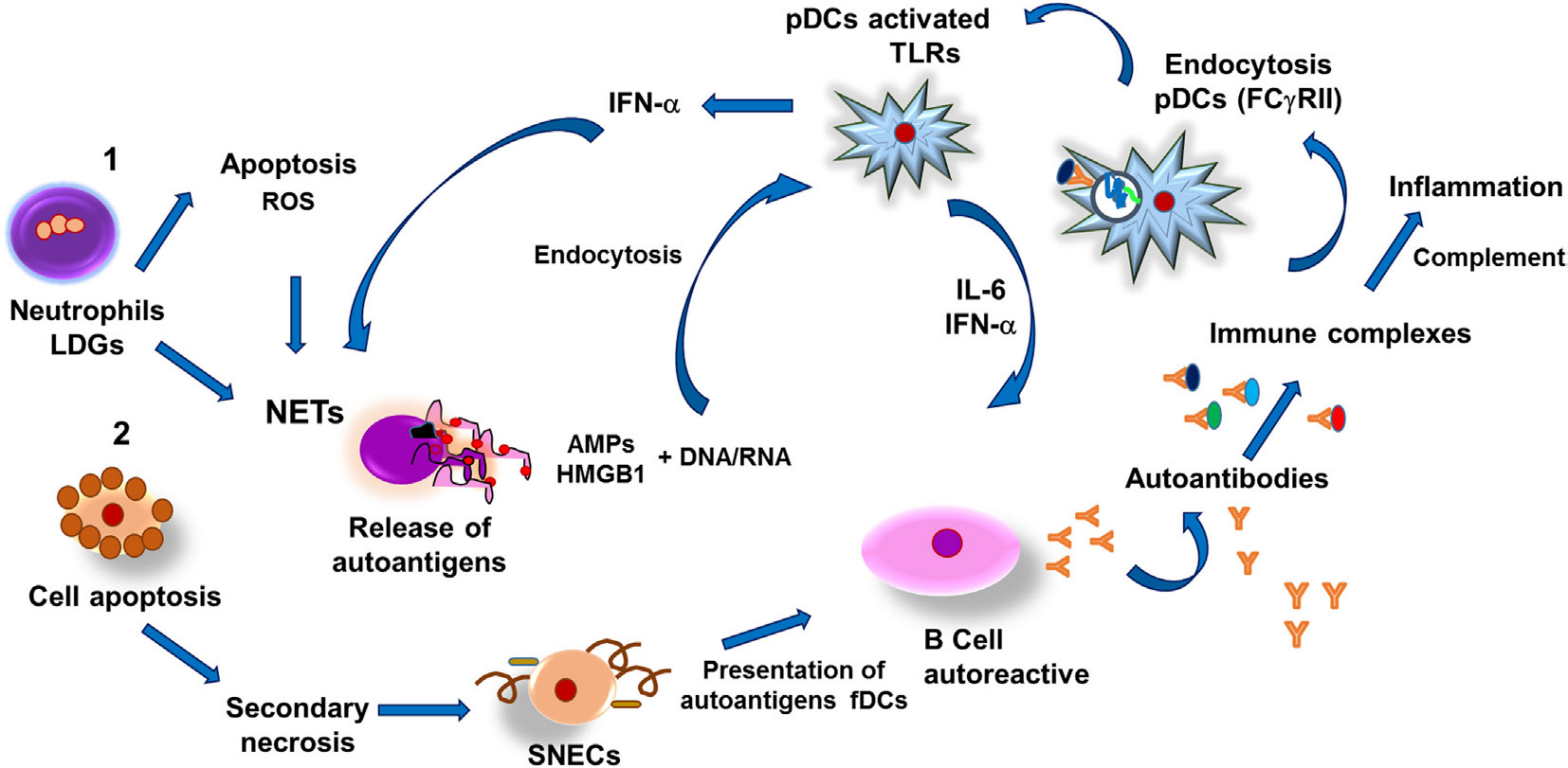
**Pathogenesis of systemic erythematosus lupus**. (1) Low-density granulocytes (LDGs) undergo apoptosis and release reactive oxygen species (ROS) and autoantigens, thus stimulating formation of neutrophil extracellular traps (NETs) as well as release of both antimicrobial peptides (AMPs) and nucleic acids (DNA, RNA). Nucleic acids and AMPs form complexes capable of binding to high-mobility group box 1 (HMGB1) protein, which can be recognized by toll-like receptors in plasmacytoid dendritic cells (pDCs), which respond by synthesizing interferon alpha (IFN-α), thus promoting formation of NETs and IL-6. Both cytokines induce differentiation of autoantibody-secreting autoreactive B cells, leading to formation of immune complexes that activate the complement system and also are susceptible to be internalized by pDCs through type II Fcγ receptors (FcγRII)-mediated endocytosis. Endosomes associate to toll-like receptors (TLRs)-containing vesicles, which results in activation of pDCs and synthesis of IFN-α, further inducing NETs and tissular inflammation. In addition, necrotic cells-derived DNA–HMGB1 complexes activate B cells resulting in production of autoantibodies and formation of immune complexes that activate pDCs, leading to IFN-α synthesis and thus establishing a positive feedback. (2) Another pathway capable of inducing autoantibodies production is mediated by release of autoantigens from apoptotic cells that undergo secondary necrosis and generate secondary necrotic cells (SNECs); accumulation of such cells in germinal centers of secondary lymphoid organs facilitates presentation of autoantigens by follicular dendritic cells (fDCs) to autoreactive B cells and subsequent formation of immune complexes that lead to persistent inflammatory process that causes tissular injury in SEL patients.

Patients with SLE have been found to possess elevated numbers of a subpopulation of neutrophils in the blood known as low-density granulocytes (LDGs). These are immature neutrophils that quickly undergo apoptosis and release ROS *in vitro*, thus acting as potent NET inducers in SLE patients (132). Through NET formation, LDG intracellular contents are released into the microenvironment and include several molecules such as LL37, α- and β-defensins and HMGB1; these molecules associate with nucleic acids and induce pDC activation though TLR9 stimulation, which subsequently induces IFN-α synthesis. It has been observed that, in SLE patients, IFN-α is a potent NETosis inducer (139) and, along with activated pDC-derived IL-6, promotes the differentiation of autoantibody-secreting autoreactive B cells (140). Another possible mechanism for the release of autoantigens such as HMGB1 and nucleic acids from apoptotic cells is through secondary necrosis, a phenomenon observed when apoptotic bodies are not properly removed by phagocytes. HMGB1 then associates with DNA and activates pDCs due to its recognition by TLR9 and receptor for advanced glycation end products (RAGE) in pDCs, thus inducing pDC activation (141). DNA–HMGB1 complexes may also be recognized by autoreactive B cells through B cell receptor–TLR7/9–RAGE; this results in the production of autoantibodies. Likewise, DNA–HMGB1–immunoglobulin may activate pDCs by interacting with RAGE–FcR–TLR9, which will lead to IFN-α synthesis and a positive feedback cycle (Figure 4) (142).

Finally, the release and persistent presence of these products represents a source of self-antigens that enhance the autoimmune and inflammatory process, leading to tissue injury in SLE.

### Rheumatoid Arthritis

Rheumatoid arthritis (RA) is a systemic autoimmune disease characterized by persistent synovial inflammation that leads to cartilage and bone injury in the joints (143). The synovial fluid at the synovial cavity of RA patients becomes infiltrated with neutrophils that readily form NETs; furthermore, even the circulating neutrophils of RA patients are more easily stimulated to NETosis than those from healthy subjects (144, 145). As occurs in other autoimmune diseases, NETs may act as a source of extracellular autoantigens; for instance, citrullinated peptides generated from histone citrullination *via* PAD2 and PAD4 activity are overexpressed in neutrophils and can be detected even in the synovia of RA patients (144, 146, 147). Such citrullinated peptides are recognized by α-citrullinated peptide antibodies (ACPAs), which form immune complexes that induce NET formation, resulting in the release of neutrophil granular contents as well as cytoplasmic self-antigens in the joints. They may also release receptor activator of nuclear factor kappa-β ligand and B-cell activating factor, which activate osteoclasts and B cells, respectively (148, 149), leading to excessive innate and adaptive immune responses in the joints and tissue injury. ACPAs are detected in the serum of RA patients at early stages of the disease and even before clinical symptoms appear, and they thus represent an early biomarker of RA (150). Khandpur et al. have found that in addition to autoantibody NET induction, IL-17 and TNF-α may also have this ability, and they found these cytokines to be elevated in RA patient serum (144).

High-mobility group box 1 is another autoantigen related to RA pathogenesis. It can be found elevated in the pannus of RA patients, particularly in the bone-cartilage interphase and areas with tissue hypoxia (151). Under hypoxic stress, cells release HMBG1 and induce production of the proinflammatory molecules TNF-α and IL-1, suggesting that HMGB1 is closely associated with hypoxia and inflammation in RA (152). Additionally, this protein binds IL-1α and IL-1β to form complexes that enhance the immune response in joints, thus provoking inflammation (141).

Some patients may develop Felty syndrome, a severe presentation of RA that manifests in patients as neutropenia and splenomegaly. The latter seems to be related to the oligoclonal expansion of T cells and autoantibodies against PAD4 (153).

### Type 1 Diabetes Mellitus

Type 1 diabetes mellitus (T1DM) is an autoimmune disease characterized by the destruction of β pancreatic cells in genetically predisposed individuals, leading to hyperglycemia. The destruction of β pancreatic cells also permits the presentation of autoantigens that are recognized by autoreactive T cells, followed by the production of specific autoantibodies for β cell antigens, including glutamic acid decarboxylase autoantibody, insulinoma-associated protein 2 autoantibody, and zinc transporter-8 autoantibody, which are used clinically as predictors of and diagnostic for T1DM, although they are not considered pathogenic (154–157).

In individuals with T1DM, infiltrates of predominantly CD8 T cells along with CD4 T cells and B cells participate in the destruction of β pancreatic cells through the release of granzymes and perforins, activation of the FasL pathway, and production of proinflammatory cytokines, namely, IFN-γ and TNF-α (158). Innate immune response cells also play an important role in the pathogenesis of T1DM, since macrophages, monocytes, DCs, and neutrophils can be found within infiltrates in pancreatic islets, wherein they synthetize IFN-α and ROS, thus promoting the synthesis of proinflammatory cytokines (154, 158, 159).

Several studies have shown that T1DM patients and individuals at risk of developing the disease suffer neutropenia (160), which may partially be attributed to increased NETosis and neutrophil infiltrate in pancreatic tissue (160–162).

Neutrophils produce superoxide and cytokines when exposed to hyperglycemic conditions. In diabetic individuals, TNF-α is elevated and activates neutrophils to form NETs and, subsequently, to release their intracellular contents, which include neutrophil serine proteases, such as neutrophil elastase (NE), PR3, and CG (161–163). T1DM patients show elevated concentrations of serum NE and PR3, as well as elevated levels of activity for these enzymes (162). These proteins are important in T1DM pathogenesis given their implication in the maturation and release of the cytokines IFN-α, IL-1β, and IL-18, as well as in inducing the expression and activation of TLRs, which are important mediators of insulitis and the destruction of pancreatic β cells (158, 164). They also favor neutrophil recruitment to sites of inflammation, providing negative feedback and contributing to pathogenesis in autoimmune diabetes (162).

### Small Vessel Vasculitis

Small vessel vasculitis (SVV) is a systemic disease of unknown etiology. SVV patients exhibit blood vessel inflammation affecting arterioles, venules, and capillaries, which may also involve arteries during disease exacerbation. In these cases, necrotizing inflammation occurs in small blood vessels and may potentially damage any organ, the primary ones being the kidneys, lungs, skin, and peripheral nerves (165). Antineutrophil cytoplasmic antibodies (ANCAs) can be detected in most SVV patients (166). Since prolonged exposure to proteins released during NETosis, such as myeloperoxidase (MPO), PR3, histones, HMGB1, and NE, is the main cause of ANCA production, these proteins are considered proinflammatory mediators that activate the complement system and lead to endothelial damage. MPO and PR3 are the principal targets of ANCAs, and it has been shown that α-PR3 and α-MPO ANCAs induce NETosis during active disease; additionally, high levels of DNA-MPO complexes are associated with disease activity (167–170). The presence of ANDA-PR3 and ANCA-MPO may activate neutrophils and perpetuate an inflammatory state through complement system activation and neutrophil chemotaxis toward the site of injury (171).

Infections may also induce the production of ANCAs through molecular mimicry. Additionally, microorganisms may induce NET formation, leading to autoantigen release (172, 173). However, since NETs have been found in SVV patients in remission, it is also important to consider that the presence of ANCAs may also help to remove NET-derived products and contribute to host homeostasis (174, 175).

Thrombosis is caused by the release of TF, cytokines, and other inflammatory mediators during NETosis occurring as a result of infection, autoimmune disease, and cancer (75, 176).

Small vessel vasculitis patients show elevated levels of NETs in the bloodstream. LDGs have been proposed as the main source of NETs due to their capacity to spontaneously generate NETs (177). These NETs may potentially amplify the inflammatory process, causing endothelial injury and activating the alternative complement pathway (170, 178). Additionally, histone presence in NETs has been demonstrated, which contributes to thrombus formation and promotes TF production, which in turn induces thrombin (165, 179). In mouse models, activated platelets have been observed to stimulate NET formation and provoke thrombosis in deep veins (180). Finally, an increased presence of neutrophil-platelet aggregates in SVV patients’ circulation has been shown to correlate to disease activity, which suggests a relationship between NETs and thrombin formation in SVV (181).

## Autoinflammatory Diseases and NETs

### Gout

**Figure 5:**
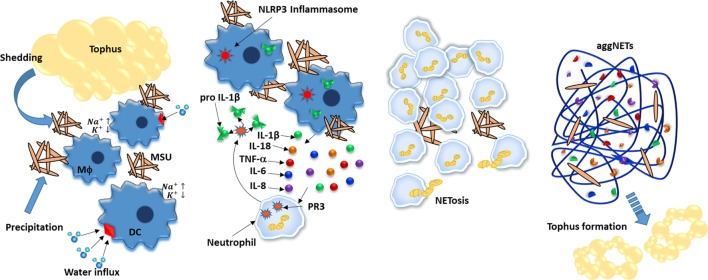
**Neutrophil extracellular traps (NETs) aliviate inflammation caused by monosodium urate (MSU) crystals**. MSU crystals are deposited in joints by precipitation and by shedding from tophus and further ingested by phagocytes such as dendritic cells and macrophages. As a consequence, the increased intracellular concentration of sodium induces cells to enhance water uptake, thus diluting potassium below inflammasome-activation concentration threshold. IL-18 and IL-1β are secreted and mediate recruitment of neutrophils to inflamed joints; neutrophils further increase IL-1b levels by cleaving pro-IL-1b in a process mediated by proteinase 3 (PR3). Upon ingesting MSU crystals, neutrophils undergo NETosis that not only degrades MSU but also encapsulates it, effectively reducing its inflammatory potential. Finally, aggregates of NETs (aggNETs) further stop inflammation by degrading *in situ* cytokines. In time, aggNETs may lead to new tophi formation.

Gout is an autoinflammatory disease characterized by the deposition of monosodium urate (MSU) crystals in the joints, which attracts leukocytes and forms structures known as tophi that mediate tissue damage. After uptake by phagocytes, MSU crystals are major stimulants of the immune response through NLRP3 inflammasome-mediated IL-1β production due to the osmotic disequilibrium caused by a sudden increase in the intracellular sodium concentration coupled with water influx and subsequent potassium dilution. Additionally, MSU-activated neutrophils secrete IL-8, TNF-α, and IL-6. These cytokines not only promote neutrophil recruitment but also induce NET formation. In particular, NETs could participate in tophi formation, since their components are closely related (Figure 5) (182, 183).

As in other diseases, NETs have been reported to promote inflammation in gout (184). However, unlike other diseases, NETs also seem to play an important role in regulating the inflammatory process and stopping gout episodes (185). Initially, NETosis reduces neutrophil density, as they indicate neutrophil death. Second, DNA nets encapsulate MSU crystals and protect them from further phagocytosis. Finally, NET-derived proteases inactivate cytokines and abrogate their proinflammatory effects (186).

### Crohn’s Disease

Crohn’s disease is a complex systemic disease that clinically manifests as gastrointestinal disorders and inflammation of the ileum and colon (187). Though inflammation is a major component of CD, the cellular components involved in its pathology remain somewhat unclear. Regarding neutrophils, their activity becomes altered. While their chemokine-mediated migration is reduced, ROS production is enhanced; furthermore, bacterial uptake seems altogether unaltered (188).

On the other hand, NET formation in CD has not been studied. Arguably, since ROS production is enhanced, neutrophils may be more prone to NET formation. Accordingly, *L. rhamnosus*, an important probiotic with both protective and corrective activity against CD, effectively inhibits NETosis (43).

### Ulcerative Colitis

Similar to CD, UC is characterized by inflammation of the gastrointestinal tract. Together, CD and UC form a clinical entity known as inflammatory bowel disease (IBD). However, unlike CD, UC is mostly restricted to colon inflammation (189, 190). Also similar to CD, the cellular components of clinical inflammation in UC are mostly unknown. Unsurprisingly, NETs have been observed in UC and correlated with inflammation by proteomic studies (191), though clearly more cellular and biochemical research is required for a clear understanding of NET involvement in IBD.

## Metabolic Diseases and NETs

It is now well known that metabolic diseases are associated with chronic low-grade inflammation driven primarily by activation of the innate immune system (192). The metabolic disorders characteristic of metabolic syndrome (MS) (hyperglycemia, hypertriglyceridemia, dyslipidemia, and hypertension) are also associated with activation of the immune system (193).

Excess caloric intake, increased fat accumulation, and lipotoxicity activate the production of effector molecules (cytokines), which in turn promote a chronic low-grade inflammatory condition that induces the recruitment and activation of many mature immune cells (including mast cells, macrophages, DCs, and neutrophils) in metabolic tissues and in adipose tissues in particular (194).

### Type 2 Diabetes

Diabetes mellitus (DM) is characterized by chronic inflammation that involves humoral factors and different types of white blood cells, including mononuclear and polymorphonuclear leukocytes. It is known that in diabetic individuals, there is an increased neutrophil count and dysfunction of phagocytic activity (195).

**Figure 6:**
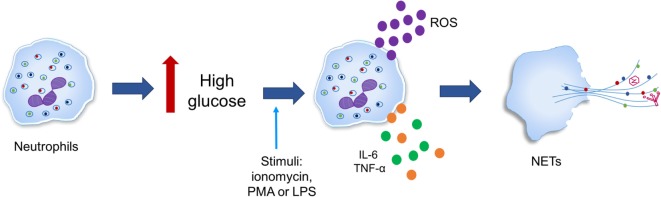
**High glucose primes neutrophils to undergo NETosis**. Neutrophils in response to inflammatory stimuli [ionomycin, phorbol-12-myristate-13-acetate (PMA), or lipopolysaccharide (LPS)] generate oxidative stress in addition to producing cytokines such as IL-6 and tumor necrosis factor alpha (TNF-α) is triggered by high glucose in type 2 diabetes.

It has been observed that the diabetic microenvironment can favor NETosis, as in diabetic conditions (hyperglycemia), neutrophils generate oxidative stress and produce cytokines such as IL-6 and TNF-α, which predispose neutrophils to produce ETs (Figure 6) (196, 197). However, hypotheses linking NETosis deregulation and hyperglycemia, oxidative stress, inflammation, and further complications of the disease remain to be confirmed (198).

Studies in humans and murine models have shown that hyperglycemia predisposes neutrophils to release NETs regardless of diabetes type when stimulated with ionomycin, PMA, or LPS. In addition, protein expression of PAD4 was found to be increased fourfold in neutrophils from individuals with diabetes when compared to those from healthy controls, suggesting that increased PAD4 may favor chromatin decondensation. However, it is still unclear whether high glucose concentrations upregulate the protein expression of PAD4 at the transcriptional or post-translational level (197).

In the sera of type 2 diabetes patients, NET-related biomarkers (elastase, mono- and oligonucleosomes, and dsDNA) are increased when compared to non-diabetic subjects; additionally, these biomarkers positively correlate with glycated hemoglobin (HbA1c) levels. In these patients, dsDNA has also been correlated with the IL-6 concentration, which may suggest a role for NETosis in the interactions between hyperglycemia and inflammation as well as in the consequences of inflammation (198).

Joshi et al. investigated whether hyperglycemic conditions could modulate NET release. They found that neutrophils exposed to high glucose concentrations and neutrophils isolated from diabetic patients had altered potential for NET release when exposed to LPS stimuli (196). Thus, they hypothesized that the chronic proinflammatory conditions present during hyperglycemia promote the constitutive formation of NETs, yet a weak response to stimuli. Fadini et al. found similar results when they analyzed NET-generation pathways in neutrophils isolated from patients with diabetic ulcers, i.e., NOX-dependent and NOX-independent pathways. In this model, neutrophils show enhanced spontaneous NETosis but a compromised capacity for induced NETosis (199).

On the other hand, neutrophils from non-diabetic individuals exposed to a high glucose concentration (25 mM) have been shown to be more susceptible to spontaneous and PMA-induced NETosis when compared to those exposed to a low glucose concentration (5 mM) and mannitol (25 mM). Since non-energetic sugars did not affect NETosis, this phenomenon could be explained by enhanced ROS production through an increase in glycolysis (198).

In summary, these data suggest that NET formation is enhanced in hyperglycemic conditions independent of diabetes type and origin.

It is important to note that in DM patients, slower wound healing represents one of the main complications. Cicatrization is a process that involves endothelial cells, fibroblasts, leukocytes, platelets, and keratinocytes. Since inflammation is a typical characteristic of the cicatrization process, neutrophils are one of the first cells recruited to the injury site, where they also act as antimicrobial cells. However, some studies report that excessive activation of NETosis-inducing neutrophils may contribute to poor wound healing (199–202).

It has been shown that PAD4-deficient mice (both diabetic and non-diabetic) possess faster wound healing and re-epithelization processes than their wild-type (WT) counterparts, independent of wound infection. This suggests that NETosis could hinder wound healing by limiting keratinocyte migration and, consequently, adequate re-epithelization. As such, NETs are a putative therapeutic target for one of the main disabling complications of diabetes (197).

Recently, it has been shown that an excess of NET-related proteins is associated with wound healing alterations and poor resolution. Furthermore, NE, oligo- and mononucleosomes, neutrophil gelatinase-associated lipocalin, and PR3 are increased in biopsies with poor healing prognosis compared to those in remission or completely healed (199).

These studies show a link between neutrophils, inflammation and tissue injury in diabetes. However, more investigation is required to fully understand the mechanisms behind NETosis and glucose metabolism.

### Obesity and MS

Obesity is characterized by an excess of adipose tissue produced as a consequence of a loss of equilibrium between energy intake and expenditure (203). The development of obesity implies a complex interaction of genetic and environmental factors that are also frequently associated with other chronic complications (hyperglycemia, dyslipidemia, hypertriglyceridemia, and high blood pressure). Individuals with three of these criteria may be clinically diagnosed as presenting MS according to the World Health Organization. MS increases the risk of developing metabolic diseases such as type 2 DM and cardiovascular diseases (194).

**Figure 7:**
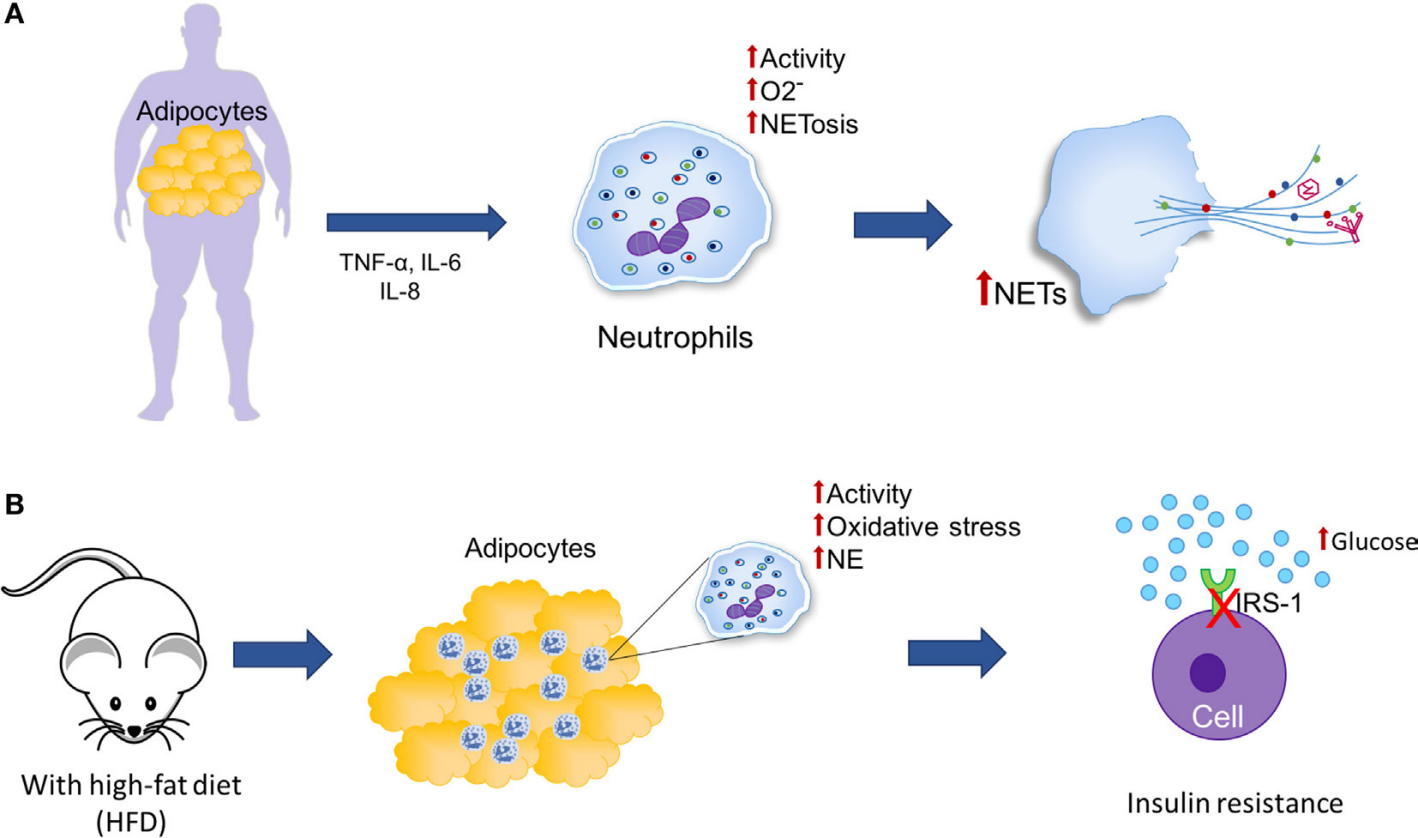
**Neutrophil extracellular traps (NETs) and adipose tissue**. **(A)** Obesity is characterized by an increase in adipose tissue and chronic low-grade inflammation in which adipocytes secrete adipokines, such as tumor necrosis factor alpha (TNF-α), IL-6, and IL-8, which have been associated with increased activity of peripheral neutrophils (generation of superoxide and induction of NETosis). **(B)** In mice under a high-fat diet (HFD), an increase in neutrophil recruitment is observed in adipose tissue and neutrophil elastase (NE) activity, consequently, it is possible that neutrophils may be promoting the insulin resistance through the degradation of insulin receptor substrate-1 (IRS-1).

Obesity has been associated with low-grade chronic inflammation in white adipose tissue (192). Adipocytes are able to secrete adipokines such as TNF-α, IL-6, and IL-8, which due to their proinflammatory properties, have been associated with enhanced activity of peripheral neutrophils, such as production of superoxide radicals and NET formation (Figure 7A). However, the effects of inflammation on adiposity and its association with NETosis are not yet clear (204–206). Since adipose tissue promotes a potentially neutrophil-activating proinflammatory environment, there exists a need to study whether the increase in adiposity may contribute to NETosis. Additionally, enhancement of glucose metabolism may lead to increments of mitochondrial-derived ROS. This phenomenon is also observed in obesity, which provokes activation of inflammatory pathways (192).

Feeding mice a high-fat diet (HFD) induces neutrophil recruitment to adipose tissue (Figure 7B). As a consequence, it is possible that neutrophils could play a role in triggering the inflammatory cascade in response to obesity (207). Accordingly, when mice fed an HFD are infected with influenza virus, viral titers are increased threefold when compared to infected mice fed a low-fat diet (LFD); the H_2_O_2_ concentration is also relatively higher in HFD mice, suggesting increased oxidative stress in their lungs. The neutrophils of these mice are more prone to spontaneous NET formation compared to neutrophils derived from LFD mice. Finally, the authors conclude that because of the elevated cytokine levels and the proinflammatory oxidative stress caused by these cytokines, adiposity associated with higher lung viral titers could represent stronger stimuli for NET formation, suggesting that in individuals with morbid obesity, lung NETs could be formed at significant levels as a response to influenza infection, thus aggravating pulmonary injury and resulting in further complications of influenza-provoked pneumonia (208).

Talukdar et al. have reported that neutrophils derived from mice fed an HFD possess higher granular content and that their NE activity is significantly higher than that of mice fed an LFD, which could promote insulin resistance through degradation of insulin receptor substrate-1 (Figure 7B). They also observed decreased insulin signaling and higher glucose production in human hepatocytes and murine adipocytes. Additionally, they showed that NE-knockout mice are more sensitive to insulin than WT mice, suggesting that the ablation of NE leads to higher hepatic insulin sensitivity and decreased expression of proinflammatory genes (207).

Oxidized low-density lipoprotein (oxLDL) is a complex mixture of LDL composed of oxidized bioactive elements with intrinsic proinflammatory activity capable of stimulating ROS production and improving the degranulation capacities of human neutrophils. Additionally, oxLDL is able to induce ROS-dependent NETosis in human neutrophils in a dose- and time-dependent manner. Interestingly, TLR2/TLR6 heterodimers seem to be necessary for oxLDL-induced NETosis as well as the PKC–IRAK–MAPK pathway, which indicated that NETosis is a multifactorial process that requires not only respiratory burst but also sequential activation of several signaling events that depend on the nature of the NET inductor. In conclusion, the inflammatory environment characterized by oxLDL and proinflammatory cytokines (IL-1β, TNF, IL-8) may be considered a potential inductor of NETs in the absence of microbial stimuli, further aggravating systemic inflammatory response syndrome, atherosclerosis and other sterile inflammatory conditions (209).

### Perspectives and Conclusion

The accumulating data on the role of neutrophils in infectious, autoimmune, autoinflammatory and metabolic diseases *via* NET structures demonstrate that they constitute novel bioindicators of prognosis and represent candidates for therapeutic targets by blocking the formation of or locally neutralizing NET signaling.

## Author Contributions

VD-R and LI-G focused on microbiological diseases related to NETs. AA-N redacted the interaction between autoimmune diseases and NETs. MM-G wrote the relationship between NETs and autoinflammatory disorders. AG-O and MF-M discussed metabolic disorders and NETs. All the authors contributed equally in the development of this review.

## Conflict of Interest Statement

The authors declare that the research was conducted in the absence of any commercial or financial relationships that could be construed as a potential conflict of interest.
